# Effects of Botulinum Toxin Type A in Essential Blepharospasm: Evidence from a Clinical and Neurophysiological Pilot Study

**DOI:** 10.3390/toxins18070276

**Published:** 2026-06-24

**Authors:** Yan Tereshko, David De Monte, Bruno Hector Ercole, Chiara Dalla Torre, Enrico Belgrado, Mariarosaria Valente, Christian Lettieri

**Affiliations:** 1 Clinical Neurology Unit, Department of Head-Neck and Neuroscience, Azienda Sanitaria Universitaria Friuli Centrale (ASUFC), 33100 Udine, Italy; 2 Department of Medicine, University of Udine, 33100 Udine, Italy; 3 Neurology Unit, Department of Head-Neck and Neuroscience, Azienda Sanitaria Universitaria Friuli Centrale (ASUFC), 33100 Udine, Italy

**Keywords:** blepharospasm, botulinum toxin type A, brainstem central effect, blink reflex recovery cycle, neurophysiological

## Abstract

Essential blepharospasm (BEB) is a focal dystonia characterized by abnormal brainstem excitability and impaired inhibitory control within trigeminal–facial circuits. Botulinum toxin type A (BoNT-A) is the established first-line treatment, primarily acting at the neuromuscular junction. However, whether BoNT-A also modulates central brainstem circuits in BEB patients remains unclear, and dedicated neurophysiological studies have yielded conflicting results. To investigate whether BoNT-A modulates brainstem interneuronal excitability in BEB using the blink reflex recovery cycle and to correlate clinical outcomes with neurophysiological results. Thirteen patients with BEB underwent neurophysiological and clinical evaluation before (T0) and one month after (T1) BoNT-A treatment. The blink reflex recovery cycle was assessed at interstimulus intervals (ISIs) of 200, 300, 500, and 1000 ms. Clinical severity was assessed using the BSPSS, JRS, and BDS scales. The R2 amplitude ratio showed a statistically significant decrease after treatment across all ISIs (all *p* ≤ 0.001), indicating reduced brainstem interneuronal excitability. All clinical scales demonstrated statistically significant improvement after treatment (BSPSS, JRS, BDS; all *p* < 0.001). This pilot study provides preliminary evidence that BoNT-A treatment may reduce brainstem interneuronal excitability in BEB patients, as evidenced by a substantial and consistent decrease in R2 amplitude ratios of the blink reflex recovery cycle. These findings are consistent with a central modulatory effect of BoNT-A beyond its established peripheral action. Larger controlled studies are warranted to confirm these results.

## 1. Introduction

Essential blepharospasm (BEB) is a focal dystonia with a prevalence that could reach 133 cases per million [1], characterized by involuntary bilateral contractions of the orbicularis oculi muscle leading to functional visual impairment and substantial reduction in quality of life. Despite its well-defined clinical features, the underlying pathophysiological mechanisms—particularly the relative contribution of peripheral and central neural dysfunction—remain incompletely understood.

Current evidence supports the involvement of basal ganglia–brainstem circuit dysfunction in BEB, resulting in impaired inhibitory control and increased excitability of brainstem interneuronal networks [2,3]. In particular, abnormal sensorimotor integration within trigeminal–facial pathways has been proposed as a key pathophysiological mechanism, leading to altered processing of afferent inputs and reduced inhibition at the brainstem level [4]. The clinical observation of sensory tricks partially suppressing spasms further supports the role of aberrant sensory processing in BEB [5].

Neurophysiological studies have provided important insights into these mechanisms. The blink reflex represents a well-established experimental model to investigate the functional integrity of trigeminal–facial circuits. The blink reflex comprises an early ipsilateral R1 response, mediated by an oligosynaptic pontine pathway, and a later bilateral R2 response, which depends on polysynaptic interneuronal circuits within the brainstem. Changes in R1 latency have also been reported in BEB, further supporting the involvement of brainstem circuits at multiple levels [2]. In addition, the recovery cycle of the R2 component is widely used to assess inhibitory processing: in healthy subjects, the second R2 response is markedly suppressed at short interstimulus intervals (ISIs), whereas in patients with BEB this suppression is reduced, reflecting increased interneuronal excitability and defective inhibitory control [6,7,8].

Botulinum toxin type A (BoNT-A) is the established first-line treatment for BEB, exerting its primary effect through inhibition of acetylcholine release at the neuromuscular junction via SNAP-25 cleavage [9]. Among BoNT-A formulations, onabotulinumtoxinA is the most widely used in clinical practice for various forms of dystonia, including BEB, with a well-established safety and efficacy profile across multiple injection cycles [10,11]. Converging experimental evidence suggests that BoNT-A effects may extend beyond the peripheral synapse. Animal studies have demonstrated retrograde axonal transport of the toxin to motor neuron cell bodies and trans-synaptic spread to second-order neurons, with documented SNAP-25 cleavage at central synaptic terminals distant from the injection site [12,13,14]. These findings raise the hypothesis that BoNT-A may modulate central neural circuits, potentially contributing to its clinical efficacy through mechanisms beyond neuromuscular blockade.

Despite these observations, whether BoNT-A modulates brainstem interneuronal excitability in BEB patients remains controversial. Valls-Solé et al. and Grandas et al. found no statistically significant changes in blink reflex recovery cycle parameters following BoNT-A treatment [15,16], whereas other groups reported partial normalization of brainstem inhibitory function [17]. These discrepancies likely reflect methodological heterogeneity, including differences in injection protocols, timing of post-treatment assessment, and—perhaps most importantly—the use of absolute R2 amplitudes rather than amplitude ratios as outcome measures, which may confound peripheral and central effects.

The present study aimed to test whether BoNT-A would reduce abnormal brainstem interneuronal excitability in BEB patients by assessing changes in the blink reflex recovery cycle before and one month after treatment. The R2 amplitude ratio was chosen as the primary outcome measure, as it normalizes for peripheral neuromuscular effects and may enable more specific detection of central modulation compared to absolute R2 amplitudes.

## 2. Results

### 2.1. Sample Characteristics

Thirteen patients with established BEB were included in the study, comprising 6 males and 7 females, with a mean age of 73.8 ± 10.7 years. All patients were on chronic BoNT-A treatment, with a mean treatment duration of 9.8 ± 6.6 years (range: 2–19 years). According to Tolosa and Martí, clinical phenotypes of BEB were classified into 3 major subtypes: clonic (n = 7), dystonic (n = 4), and tonic (n = 2) [18]. Eyelid opening apraxia (EOA) was present in 5 patients (38.5%: 2 clonic, 2 dystonic, 1 tonic). Demographic and clinical characteristics are summarized in Table 1.

### 2.2. Neurophysiological Findings

Neurophysiological outcomes were analyzed separately for the right eye (Table 2), the left eye (Table 3), and both eyes combined (Table 4), with the single-eye analyses representing the primary neurophysiological outcome and the combined analysis included as a supplementary analysis given its greater statistical power. Following BoNT-A treatment, a statistically significant reduction in both unconditioned and conditioned R2 amplitudes was observed across all ISIs and both sides (all *p* < 0.05, Table 2, Table 3 and Table 4), consistent with the known peripheral neuromuscular effect of the toxin. Of note, the R2 amplitude ratio showed a statistically significant decrease after treatment at ISIs of 200, 300, and 500 ms when each eye was analyzed separately, with the effect at ISI 1000 ms reaching statistical significance only for the left eye (*p* = 0.010) but not the right eye (*p* = 0.052). When both eyes were considered together, the reduction in amplitude ratio was statistically significant across all ISIs (all *p* ≤ 0.001), providing stronger evidence of reduced brainstem interneuronal excitability following treatment.

**Figure 1:**
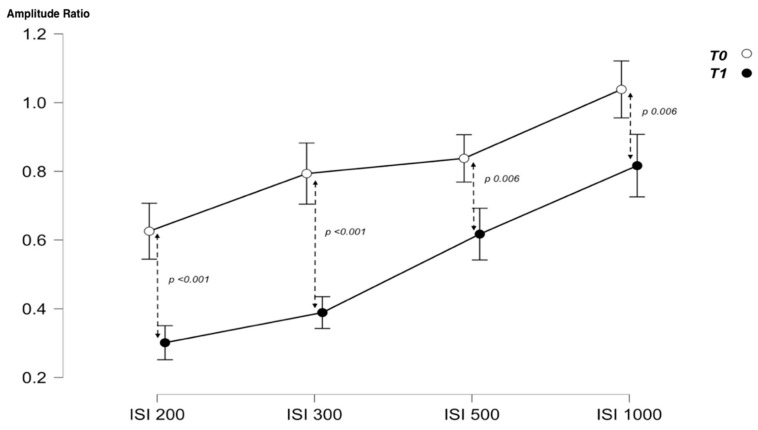
Plot line (with 95% confidence intervals) of the amplitude ratio at different paired-pulse ISIs (200 ms, 300 ms, 500 ms, and 1000 ms), considering both eyes. The repeated measures ANOVA analysis confirmed a statistically significant increase in the amplitude ratio with the increase in the ISI, both at the baseline (T0) and one month after the treatment (T1) with botulinum toxin type A (F_2.766,138.320_ = 59.886, *p* < 0.001, η^2^ = 0.310). There was also a statistically significant amplitude ratio difference between the baseline and one month after the treatment (F_1,50_ = 58.997, *p* < 0.001, η^2^ = 0.225).

**Figure 2:**
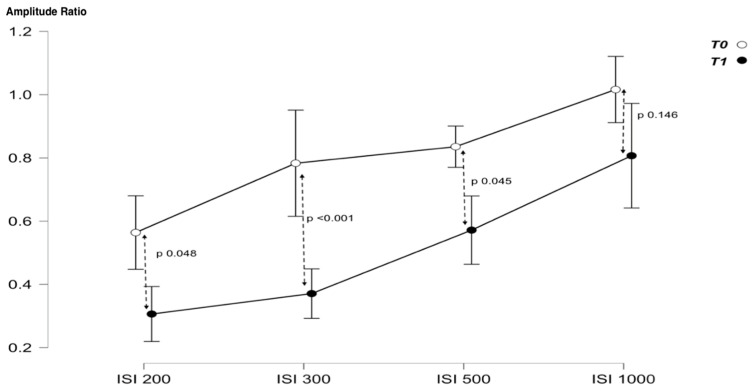
Plot line (with 95% confidence intervals) of the amplitude ratio at different paired-pulse ISIs (200 ms, 300 ms, 500 ms, and 1000 ms), considering the right eye. The repeated measures ANOVA analysis confirmed a statistically significant increase in the amplitude ratio with the increase in the ISI, both at the baseline (T0) and one month after the treatment (T1) with botulinum toxin type A (F_2.524,60.574_ = 28.330, *p* < 0.001, *η*^2^ = 0.316). There was also a statistically significant amplitude ratio difference between the baseline and one month after the treatment (F_1,24_ = 26.293, *p* < 0.001, *η*^2^ = 0.210).

**Figure 3:**
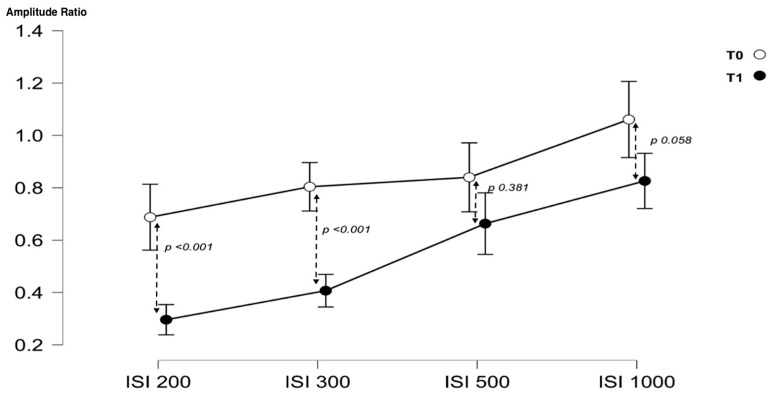
Plot line (with 95% confidence intervals) of the amplitude ratio at different paired-pulse ISIs (200 ms, 300 ms, 500 ms, and 1000 ms), considering the left eye. The repeated measures ANOVA analysis confirmed a statistically significant increase in the amplitude ratio with the increase in the ISI, both at the baseline (T0) and one month after the treatment (T1) with botulinum toxin type A (F_2.524,60.574_ = 30.401, *p* < 0.001, η^2^ = 0.308). There was also a statistically significant amplitude ratio difference between the baseline and one month after the treatment (F_1,24_ = 32.077, *p* < 0.001, η^2^ = 0.243).

Repeated measures ANOVA confirmed a statistically significant effect of treatment on R2 ratios when considering both eyes together (F_1,50_ = 58.997, *p* < 0.001, η^2^ = 0.225), as well as for the right eye alone (F_1,24_ = 26.293, *p* < 0.001, η^2^ = 0.210) and the left eye alone (F_1,24_ = 32.077, *p* < 0.001, η^2^ = 0.243). A statistically significant increase in amplitude ratio with increasing ISI was confirmed at both timepoints (F_2.766,138.320_ = 59.886, *p* < 0.001, η^2^ = 0.310), with the T1 curve consistently below the T0 curve across all ISIs, reflecting a consistent reduction in brainstem interneuronal excitability following treatment (Figure 1). A similar pattern was observed when each side was analyzed separately (Figure 2 and Figure 3). However, following Bonferroni correction, statistical significance was lost at ISI 1000 ms for the right eye (*p* = 0.052) and at ISI 500 ms for the left eye (*p* = 0.061), indicating that the central modulatory effect of BoNT-A is most consistently expressed at shorter interstimulus intervals.

### 2.3. Clinical Outcomes

All patients showed clinical improvement following BoNT-A treatment. Statistically significant reductions were observed in all three clinical scales at T1 compared to T0: BSPSS decreased from 7.6 ± 1.9 to 5.4 ± 1.7 (*p* < 0.001), JRS from 5.9 ± 1.3 to 3.4 ± 1.3 (*p* < 0.001), and BDS from 12.1 ± 5.8 to 6.5 ± 4.9 (*p* < 0.001), as detailed in Table 5.

### 2.4. Subgroup Analyses

**Figure 4:**
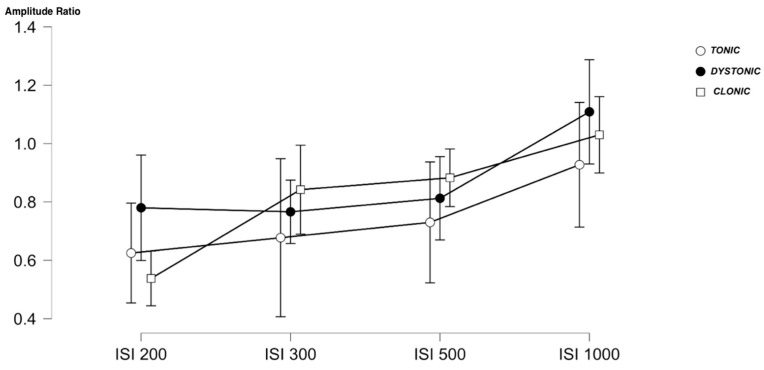
Plot line (with 95% confidence intervals) of the amplitude ratio at different paired-pulse ISIs (200 ms, 300 ms, 500 ms, and 1000 ms) obtained at the baseline for the clonic, tonic, and dystonic blepharospasm subtypes. There was no statistically significant difference between them (F_2,23_ = 0.724, *p* = 0.496, η^2^ = 0.023).

**Figure 5:**
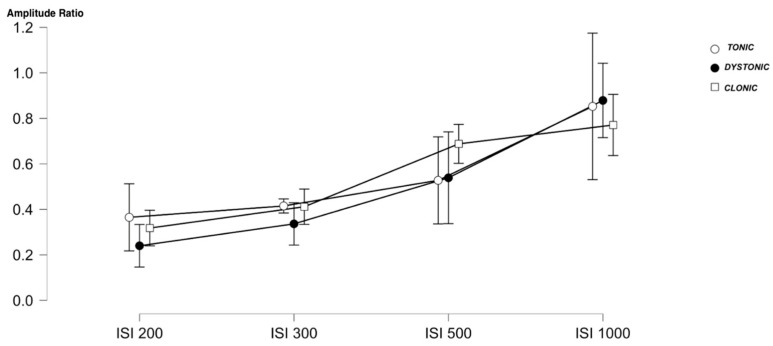
Plot line (with 95% confidence intervals) of the amplitude ratio at different paired-pulse ISIs (200 ms, 300 ms, 500 ms, and 1000 ms) obtained one month after the treatment with botulinum toxin type A for the clonic, tonic, and dystonic blepharospasm subtypes. There was no statistically significant difference between them (F_2,23_ = 0.686, *p* = 0.514, η^2^ = 0.008).

All subgroup analyses are exploratory and should be interpreted with caution, given the small group sizes and the inherent risk of both type I and type II error. No statistically significant differences in R2 amplitude ratios were observed across clinical subtypes (clonic, dystonic, and tonic) either at baseline (F_2,23_ = 0.724, *p* = 0.496, η^2^ = 0.023, Figure 4) or one month after treatment (F_2,23_ = 0.686, *p* = 0.514, η^2^ = 0.008, Figure 5), suggesting that brainstem excitability is similarly modulated by BoNT-A regardless of the clinical phenotype. However, the reduction in JRS score observed in the tonic subgroup was significantly smaller compared to both the clonic (1.0 ± 1.2 vs. 2.7 ± 0.7, *p* = 0.018) and dystonic (1.0 ± 1.2 vs. 3.0 ± 1.3, *p* = 0.010) subgroups (F_2,10.389_ = 4.536, *p* = 0.038, η^2^ = 0.337). This finding should be interpreted with caution, given the very small size of the tonic subgroup (n = 2). No statistically significant differences were observed in BSPSS (F_2,14.171_ = 1.997, *p* = 0.172, η^2^ = 0.148) or BDS (F_2,10.484_ = 1.006, *p* = 0.398, η^2^ = 0.085) reduction across subtypes.

**Figure 6:**
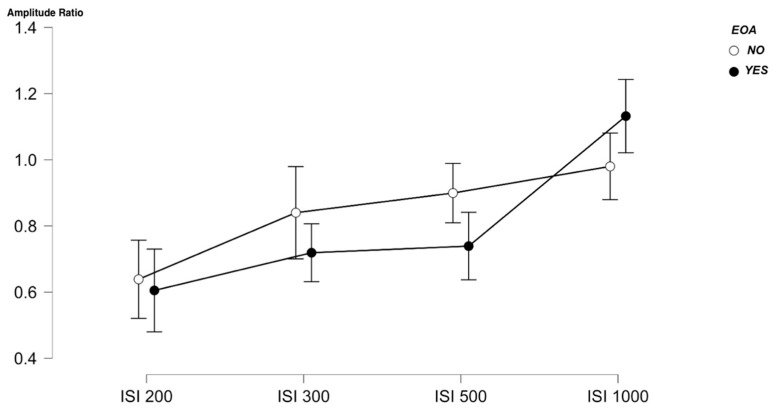
Plot line (with 95% confidence intervals) of the amplitude ratio at different paired-pulse ISIs (200 ms, 300 ms, 500 ms, and 1000 ms) obtained at the baseline for the patient with and without concomitant EOA. There was no statistically significant difference between them (F_1,24_ = 0.344, *p* = 0.563, η^2^ = 0.005).

**Figure 7:**
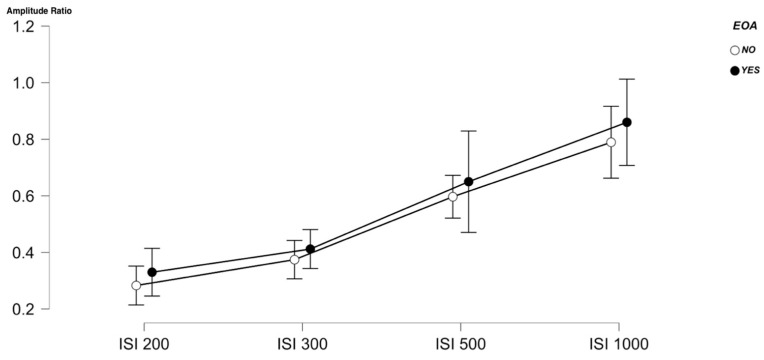
Plot line (with 95% confidence intervals) of the amplitude ratio at different paired-pulse ISIs (200 ms, 300 ms, 500 ms, and 1000 ms) obtained one month after the treatment of the patients with and without concomitant EOA. There was no statistically significant difference between them (F_1,24_ = 1.969, *p* = 0.173, η^2^ = 0.009).

Patients with EOA (n = 5) showed no statistically significant differences in R2 amplitude ratios compared to those without EOA, either at baseline (F_1,24_ = 0.344, *p* = 0.563, η^2^ = 0.005, Figure 6) or after treatment (F_1,24_ = 1.969, *p* = 0.173, η^2^ = 0.009, Figure 7). Similarly, no statistically significant differences were observed between EOA and non-EOA patients in clinical scale reductions (BSPSS: F_2,13.389_ = 0.006, *p* = 0.939; JRS: F_2,14.767_ = 1.172, *p* = 0.296; BDS: F_2,15.741_ = 0.020, *p* = 0.980).

## 3. Discussion

The main finding of this study is that BoNT-A treatment appears to reduce brainstem interneuronal excitability in patients with BEB, as evidenced by a decrease in R2 amplitude ratios of the blink reflex recovery cycle, particularly at shorter interstimulus intervals. Critically, this reduction in amplitude ratios was observed independently of the expected peripheral neuromuscular effect of the toxin, supporting a direct central modulatory action of BoNT-A. The blink reflex recovery cycle is a well-established measure of inhibitory processing within trigeminal–facial brainstem circuits [8,19]. In patients with BEB, reduced suppression of the second R2 response at short ISIs reflects increased interneuronal excitability and impaired inhibitory control—a well-established neurophysiological feature of the condition [2,8,15]. The observed reduction in R2 amplitude ratios following BoNT-A treatment indicates a substantial, though not complete, reduction in brainstem interneuronal excitability. At T0, R2 amplitude ratios observed in this cohort substantially exceeded those reported in healthy subjects [8,20], suggesting brainstem hyperexcitability at baseline, though direct comparison with an internal control group recorded with the same protocol was not possible. Following treatment, T1 values showed a substantial reduction, remaining above published normative values, particularly at shorter ISIs. This pattern of improvement is consistent with the clinically meaningful but incomplete improvement observed in clinical scales.

The decrease in both conditioned and unconditioned R2 amplitudes is consistent with the established peripheral mechanism of action of BoNT-A—inhibition of acetylcholine release at the neuromuscular junction through SNAP-25 cleavage [9]. However, the concomitant and statistically significant reduction in R2 amplitude ratios, which normalize for peripheral neuromuscular effects, provides preliminary evidence of a central modulatory action beyond the neuromuscular junction. A plausible mechanism demonstrated in animal models is retrograde axonal transport of BoNT-A to motor neuron cell bodies, followed by trans-synaptic spread to second-order neurons, with consequent SNAP-25 cleavage at central synaptic terminals [12,13,21]—though direct evidence in humans remains lacking. This would result in reduced neurotransmitter release within brainstem interneuronal circuits, thereby decreasing excitability of trigeminal–facial pathways, consistent with the reduction in R2 amplitude ratios observed in the present study.

Previous studies investigating the effects of BoNT-A on the blink reflex recovery cycle have reported conflicting results. The discrepancy between the present findings and those of Valls-Solé et al. and Grandas et al., who reported no statistically significant changes in brainstem excitability following BoNT-A treatment, can be attributed to several methodological differences. First, both prior studies assessed patients at approximately three weeks post-injection, whereas in the present study, patients were assessed one month after treatment—a timeframe more consistent with the time course of both peripheral and central neuromodulatory effects of BoNT-A. Experimental studies have demonstrated that central SNAP-25 cleavage in the facial nucleus following peripheral BoNT-A injection increases progressively over several weeks after injection [12,13,21]. Furthermore, the maximum peripheral effect at the neuromuscular junction has been reported to occur at approximately four weeks after injection [22], suggesting that one month may better reflect the full expression of both peripheral and central effects of BoNT-A, and may be more appropriate than the three-week assessment used in prior studies. Second, Grandas et al. employed unilateral injections and recorded exclusively from the untreated side, introducing potential confounders related to inter-side asymmetry. Third, and perhaps most critically, previous studies primarily relied on absolute R2 amplitudes, which are substantially influenced by the peripheral neuromuscular effect of BoNT-A. In contrast, the R2 amplitude ratio—used in the present study as the primary outcome—normalizes for peripheral chemodenervation, providing a more specific index of brainstem interneuronal excitability. The statistically significant reduction in R2 amplitude ratios observed in this cohort therefore supports a direct central modulatory effect, independent of peripheral muscle weakness.

When each side was analyzed separately, the reduction in R2 amplitude ratio was most robust at shorter ISIs (200–500 ms), with statistical significance at ISI 1000 ms being less consistent after Bonferroni correction. This pattern likely reflects two concurrent factors: first, at longer ISIs, the degree of R2 suppression decreases physiologically even in healthy subjects, resulting in greater inter-individual variability and reduced sensitivity to detect treatment-related changes; second, the difference in R2 amplitude ratios between T0 and T1 is smaller at longer ISIs, requiring a larger sample size to reach statistical significance. When both eyes were analyzed together, the treatment effect was statistically significant across all ISIs, consistent with the single-eye analyses and providing further convergent evidence of a treatment-related reduction in brainstem interneuronal excitability following BoNT-A treatment. No statistically significant differences in R2 amplitude ratios were observed across clinical subtypes or between patients with and without EOA, suggesting that BoNT-A exerts a similar modulatory effect on brainstem interneuronal excitability regardless of the clinical phenotype or the presence of apraxia. This finding may indicate that the central neuromodulatory mechanism of BoNT-A acts within the trigeminal–facial circuit common to all BEB phenotypes, regardless of the clinical presentation. The reduction in JRS score observed in the tonic subgroup was significantly smaller compared to both the clonic and dystonic phenotypes. Whether this reflects a genuine difference in clinical responsiveness to BoNT-A across subtypes or rather a limitation of the JRS in capturing improvement in the tonic phenotype remains unclear and warrants investigation in larger cohorts. The subgroup analyses conducted in this pilot study are inherently exploratory, and no subgroup-specific conclusions can be drawn from the present data.

The absence of neurophysiological differences in patients with EOA is noteworthy, as EOA has been proposed to involve distinct pathophysiological mechanisms, including impaired voluntary eyelid opening due to supranuclear or cortical dysfunction [23]. The lack of a distinct neurophysiological signature in the EOA subgroup may suggest that brainstem intraneuronal excitability, as measured by the blink reflex recovery cycle, does not specifically capture the mechanisms underlying EOA—or perhaps that EOA does not confer additional brainstem hyperexcitability beyond that already present in BEB.

In the present cohort, no statistically significant correlation was observed between the reduction in R2 amplitude ratios and the degree of clinical improvement in any of the rating scales assessed. This finding may reflect the small sample size and the consequent limited statistical power to detect moderate correlations, rather than a true absence of a relationship between neurophysiological and clinical outcomes. Future studies with larger cohorts should specifically investigate this association, as a strong correlation would provide direct evidence linking central neuromodulation to the therapeutic efficacy of BoNT-A in BEB and could potentially serve as a neurophysiological biomarker of treatment response.

### Limitations of the Study

Several limitations of this study should be acknowledged. The small sample size (n = 13) reduces statistical power and limits the generalizability of the findings, particularly for subgroup analyses—the tonic subgroup (n = 2) is too small to draw any meaningful phenotype-specific conclusions—and larger multicenter studies are needed to confirm and extend these results. No formal a priori power calculation was performed given the pilot design of the study; however, the observed effect sizes for the primary neurophysiological outcome were large for both the right eye (η^2^ = 0.210, F_1,24_ = 26.293) and left eye (η^2^ = 0.243, F_1,24_ = 32.077) analyses, exceeding the conventional threshold for large effects (η^2^ > 0.14), suggesting that the study had adequate sensitivity to detect the primary treatment effect at the observed sample size. These values are comparable to effect sizes observed in analogous neurophysiological studies on brainstem excitability in BEB conducted on similar or smaller sample sizes [15,16], supporting the statistical adequacy of the pilot design for the detection of the primary outcome.

The bilateral analysis (n = 26) did not formally account for within-subject correlation between eyes, as each eye was treated as an independent observation; this represents a statistical limitation, and the single-eye analyses (n = 13) should be considered the primary neurophysiological outcome. Additionally, the R2 amplitude ratio may not fully correct for peripheral neuromuscular effects, as BoNT-A could potentially alter the morphology of the peripheral EMG response, including temporal dispersion and synchronization of muscle fiber activation.

The absence of a healthy control group and an untreated BEB control group limits the interpretation of the neurophysiological changes observed, since spontaneous fluctuations in brainstem excitability over time cannot be excluded as a contributing factor to the observed reduction in R2 amplitude ratios; moreover, the lack of an internal normative dataset acquired with the same protocol precludes definitive conclusions regarding normalization of brainstem excitability, and the observed changes should therefore be interpreted as treatment-related reductions in blink reflex recovery parameters.

Only a single post-treatment timepoint was assessed, leaving the onset, peak, and duration of the central modulatory effect of BoNT-A uncharacterized, and longitudinal assessments at multiple timepoints would be needed to fully elucidate its time course. All patients were already on chronic BoNT-A treatment prior to enrolment, and the potential cumulative neuromodulatory effects of repeated injections could not be quantified, leaving open the question of whether the observed changes reflect an acute or long-term effect of treatment. Finally, the single-center design and the use of a standardized injection protocol may further limit the generalizability of these findings to settings where different toxin formulations, doses, or techniques are used.

## 4. Conclusions

This pilot study provides preliminary evidence that BoNT-A treatment may reduce brainstem interneuronal excitability in patients with BEB, as evidenced by a substantial and consistent decrease in R2 amplitude ratios of the blink reflex recovery cycle. The concomitant improvement in clinical outcomes supports the hypothesis that the therapeutic efficacy of BoNT-A in BEB depends not only on peripheral chemodenervation but also on central neuromodulation of brainstem inhibitory circuits—a mechanism consistent with retrograde axonal transport and trans-synaptic spread of the toxin, as demonstrated in animal models. These findings contribute to a better understanding of both the mechanism of action of botulinum toxin and the pathophysiology of BEB. Future studies with larger cohorts, healthy control groups, multiple follow-up timepoints, and direct correlation analyses between neurophysiological and clinical outcomes are warranted to confirm and extend these results.

## 5. Materials and Methods

### 5.1. Study Outcomes

#### 5.1.1. Primary Outcome Measure

The primary outcome measure was the R2 amplitude ratio (conditioned R2/unconditioned R2) of the blink reflex recovery cycle at interstimulus intervals of 200, 300, 500, and 1000 ms, assessed before (T0) and one month after (T1) BoNT-A treatment, with the aim of determining whether treatment induces a measurable change in brainstem interneuronal excitability in patients with BEB.

#### 5.1.2. Secondary Outcome Measures

The secondary outcome measures were: (i) to evaluate the effect of BoNT-A on the absolute amplitude of the conditioned and unconditioned R2 responses; (ii) to assess changes in clinical severity using validated rating scales before and after treatment; (iii) to compare R2 amplitude ratios and absolute R2 amplitudes across clinical subtypes of blepharospasm (clonic, dystonic, and tonic); and (iv) to investigate whether the presence of eyelid opening apraxia influences neurophysiological or clinical outcomes.

### 5.2. Participants

A total of 13 patients with an established diagnosis of BEB already undergoing chronic BoNT-A treatment were enrolled at the Clinical Neurology Unit, University of Udine, between December 2023 and November 2024. Diagnosis had been established by a movement disorders specialist according to standard clinical criteria. All patients had discontinued BoNT-A treatment for at least 5 months prior to baseline assessment (T0), ensuring return to baseline disease state before re-evaluation. Inclusion criteria were: (i) established diagnosis of BEB and (ii) eligibility for re-treatment with BoNT-A. Exclusion criteria included: (i) secondary forms of blepharospasm; (ii) other neurological disorders affecting brainstem function; and (iii) use of medications potentially interfering with neurophysiological assessments, including anticholinergics, benzodiazepines, and antispastic agents.

### 5.3. Study Design

This was a longitudinal observational study. Patients were evaluated at baseline (T0), immediately before BoNT-A injection, and at follow-up (T1), one month after treatment. The one-month timepoint was chosen as it corresponds to the peak clinical effect of BoNT-A and allows sufficient time for potential central neuromodulatory effects to manifest, consistent with experimental evidence on the time course of retrograde axonal transport [12].

### 5.4. Botulinum Toxin Treatment

All patients were treated with onabotulinumtoxinA injections administered by an experienced neurologist. The injection protocol targeted the pretarsal portion of the orbicularis oculi muscles bilaterally, with additional injections into the orbital portion of the orbicularis oculi and the corrugator supercilii muscles when clinically indicated [24]. The total dose per patient was 46.9 ± 13.2 (range: 26–68 U). Injections were performed using anatomical landmarks. All patients had discontinued BoNT-A treatment for at least 5 months prior to baseline assessment.

### 5.5. Neurophysiological Assessment

Blink reflex recordings were performed in all patients at T0 and T1 under standardized conditions, with patients seated at rest in a quiet room, using a Dantec Keypoint^®^ G4 electromyography system (Natus Medical Incorporated, Middleton, WI, USA) with the following parameters: bandpass filter 2 Hz–10 kHz, gain 0.2 mV/div, and sweep speed 5 ms/div. Electrical stimulation was applied bilaterally to the supraorbital nerve using surface electrodes, with supramaximal stimulus intensity (up to 30 mA) and pulse duration of 0.2 ms, delivered at a randomized stimulation rate, in accordance with established neurophysiological methodology for blink reflex assessment [25]. Recordings were obtained bilaterally from the orbicularis oculi muscles. Muscle relaxation was verified prior to each trial by confirming isoelectric baseline EMG activity; trials with evidence of pre-activation or spontaneous orbicularis oculi activity were excluded and repeated. The blink reflex recovery cycle was assessed using paired stimuli delivered at ISIs of 200, 300, 500, and 1000 ms. For each ISI, two trials were recorded and averaged—a number considered sufficient to verify response reproducibility while minimizing the risk of habituation of the R2 component. Peak-to-peak amplitude measurements of the R2 component were performed using automated analysis with manual correction by an experienced neurophysiologist blinded to both the patients’ clinical scores and the recording timepoint (T0 vs. T1). The R2 amplitude ratio was calculated as the ratio of the conditioned R2 amplitude to the unconditioned R2 amplitude.

### 5.6. Clinical Assessment

Clinical severity was evaluated at T0 and T1 using three validated rating scales. The Blepharospasm Severity Rating Scale (BSPSS) assesses the frequency and intensity of spasms. The Jankovic Rating Scale (JRS) evaluates both severity and frequency of dystonic movements. The Blepharospasm Disability Scale (BDS) quantifies functional impairment in daily activities. All clinical assessments were performed by a movement disorders specialist blinded to neurophysiological results.

### 5.7. Statistical Analysis

The descriptive analysis of the sample was performed using mean ± SD for continuous variables and frequencies for categorical variables. A Shapiro–Wilk test was used to assess the normal distribution of data. A *t*-test, or a Mann–Whitney test if the data distribution was not normal, was used to compare continuous variables. Repeated measures ANOVA was performed to investigate the changes and the differences between the R2 amplitude ratios obtained at different ISIs (200, 300, 500, and 1000 ms) at baseline (T0) and one month after BoNT-A treatment (T1). Since Mauchly’s test indicated a violation of sphericity, the Greenhouse–Geisser correction was applied. The Bonferroni post hoc test was used to compare means at different timepoints. The dataset was complete, with no missing values. All analyses were performed using Stata/SE (version 15.1, StataCorp, College Station, TX, USA). All two-tailed significance levels were set at *p* < 0.05. No formal a priori sample size calculation was performed, as this study was designed as a pilot study. All consecutive patients with BEB eligible for BoNT-A re-treatment at the enrolling center during the study period were prospectively included, and the findings should be interpreted in the context of this sample size.

### 5.8. Ethics

This study was conducted in accordance with the Declaration of Helsinki and approved by the Institutional Review Board of the University of Udine (Protocol no. 19/2014). All patients provided written informed consent for treatment and for the use of their clinical data for research purposes.

## Figures and Tables

**Table 1:** Demographic data of the studied population. Quantitative data are reported as mean (SD), and qualitative data are reported as frequencies.

	Demographic Data
Sex (male/female)	6/7
Age (years)	73.8 ± 10.7 (range 55–88)
Disease duration (years) Clinical phenotypes	9.8 ± 6.6
- Tonic	2/13
- Clonic	7/13
- Dystonic	4/13
Eyelid opening apraxia (EOA)	5/13

**Table 2:** Comparison between the amplitude of the unconditioned R2, conditioned R2, and amplitude ratio obtained in the right eye at the baseline and 1 month after the treatment with botulinum toxin. A *t*-test or a Mann–Whitney test was used, as appropriate, to compare continuous variables.

Right Eye (n = 13)	T0	T1	*p*	Effect Size	95% CI (Effect Size)
					
ISI 200					
Amp R2 uncon	0.192 ± 0.135	0.095 ± 0.051	0.006	0.824	0.503–0.954
Amp R2 cond	0.097 ± 0.051	0.026 ± 0.012	<0.001	1.000	1.000–1.000
Ratio	0.564 ± 0.192	0.360 ± 0.146	0.002	0.710	0.409–0.872
					
ISI 300					
Amp R2 uncon	0.217 ± 0.128	0.119 ± 0.078	0.031	0.718	0.257–0.913
Amp R2 cond	0.127 ± 0.108	0.041 ± 0.024	<0.001	1.000	1.000–1.000
Ratio	0.783 ± 0.369	0.371 ± 0.140	0.001	0.740	0.460–0.886
					
ISI 500					
Amp R2 uncon	0.159 ± 0.09	0.100 ± 0.067	0.014	0.780	0.405–0.931
Amp R2 cond	0.134 ± 0.086	0.055 ± 0.044	<0.001	1.000	1.000–1.000
Ratio	0.835 ± 0.194	0.572 ± 0.193	0.003	0.680	0.361–0.857
					
ISI 1000					
Amp R2 uncon	0.155 ± 0.087	0.105 ± 0.079	0.059	0.579	−0.021–1.159
Amp R2 cond	0.156 ± 0.093	0.084 ± 0.072	0.025	0.713	0.089–1.314
Ratio	1.016 ± 0.190	0.807 ± 0.248	0.013	0.580	0.207–0.806

**Table 3:** Comparison between the amplitude of the unconditioned R2, conditioned R2, and amplitude ratio obtained in the left eye at the baseline and 1 month after the treatment with botulinum toxin. A *t*-test or a Mann–Whitney test was used, as appropriate, to compare continuous variables.

Left Eye (n = 13)	T0	T1	*p*	Effect Size	95% CI (Effect Size)
					
ISI 200					
Amp R2 uncon	0.227 ± 0.117	0.115 ± 0.074	<0.001	1.244	0.497–1.963
Amp R2 cond	0.154 ± 0.094	0.033 ± 0.021	<0.001	1.000	1.000–1.000
Ratio	0.688 ± 0.251	0.296 ± 0.089	<0.001	0.935	0.847–0.973
					
ISI 300					
Amp R2 uncon	0.217 ± 0.128	0.119 ± 0.078	0.031	0.718	0.257–0.913
Amp R2 cond	0.174 ± 0.114	0.037 ± 0.030	0.002	1.000	1.000–1.000
Ratio	0.804 ± 0.208	0.407 ± 0.109	<0.001	1.000	1.000–1.000
					
ISI 500					
Amp R2 uncon	0.223 ± 0.142	0.108 ± 0.074	0.005	0.963	0.286–1.614
Amp R2 cond	0.186 ± 0.136	0.071 ± 0.050	0.002	0.912	0.728–0.974
Ratio	0.840 ± 0.223	0.663 ± 0.230	0.073	0.402	−0.004–0.716
					
ISI 1000					
Amp R2 uncon	0.197 ± 0.134	0.088 ± 0.051	0.004	0.968	0.290–1.619
Amp R2 cond	0.191 ± 0.092	0.071 ± 0.040	<0.001	0.978	0.927–0.994
Ratio	1.061 ± 0.282	0.826 ± 0.181	0.026	0.521	0.124–0.774

**Table 4:** Comparison between the amplitude of the unconditioned R2, conditioned R2, and amplitude Ratio, obtained considering both eyes, at the baseline and 1 month after the treatment with botulinum toxin. A *t*-test or a Mann–Whitney test was used, as appropriate, to compare continuous variables.

Both Eyes (n = 26)	T0	T1	*p*	Effect Size	95% CI (Effect Size)
					
ISI 200					
Amp R2 uncon	0.210 ± 0.125	0.105 ± 0.063	<0.001	0.575	0.326–0.750
Amp R2 cond	0.126 ± 0.080	0.030 ± 0.017	<0.001	0.873	0.773–0.930
Ratio	0.626 ± 0.228	0.301 ± 0.119	<0.001	0.824	0.692–0.903
					
ISI 300					
Amp R2 uncon	0.196 ± 0.124	0.118 ± 0.081	0.008	0.432	0.144–0.652
Amp R2 cond	0.150 ± 0.111	0.039 ± 0.026	<0.001	0.849	0.733–0.917
Ratio	0.793 ± 0.294	0.389 ± 0.124	<0.001	0.877	0.781–0.933
					
ISI 500					
Amp R2 uncon	0.191 ± 0.121	0.104 ± 0.069	0.002	0.513	0.245–0.708
Amp R2 cond	0.160 ± 0.114	0.063 ± 0.047	<0.001	0.660	0.443–0.804
Ratio	0.838 ± 0.205	0.617 ± 0.213	<0.001	0.550	0.293–0.733
					
ISI 1000					
Amp R2 uncon	0.176 ± 0.113	0.096 ± 0.065	<0.001	0.493	0.219–0.694
Amp R2 cond	0.173 ± 0.092	0.078 ± 0.057	<0.001	0.663	0.447–0.806
Ratio	1.038 ± 0.237	0.817 ± 0.213	0.001	0.527	0.262–0.717
					

**Table 5:** Comparison between the BSPSS, JRS, and BDS obtained at the baseline and 1 month after the treatment with botulinum toxin. A *t*-test or a Mann–Whitney test was used, as appropriate, to compare continuous variables.

N = 13	T0	T1	*p*	Effect Size	95% CI (Effect Size)
BSPSS	7.6 ± 1.9	5.4 ± 1.7	<0.001	1.567	0.729–2.378
JRS	5.9 ± 1.3	3.4 ± 1.3	<0.001	2.118	1.108–3.104
BDS	12.1 ± 5.8	6.5 ± 4.9	<0.001	1.484	1.000–1.000

## Data Availability

The original contributions presented in this study are included in the article. Further inquiries can be directed to the corresponding author(s).

## Abbreviations

BDS: Blepharospasm Disability Scale; BEB: Essential Blepharospasm; BoNT-A: Botulinum Toxin Type A; BSPSS: Blepharospasm Severity Rating Scale; EOA: Eyelid Opening Apraxia; ISI: Interstimulus Interval; JRS: Jankovic Rating Scale; R1: First component of the blink reflex; R2: Second component of the blink reflex; SNAP-25: Synaptosomal-Associated Protein 25; T0: Baseline assessment; T1: Follow-up assessment at one month.
